# Analysis of *BRCA* Germline Mutations in Chinese Prostate Cancer Patients

**DOI:** 10.3389/fonc.2022.746102

**Published:** 2022-02-17

**Authors:** Wei Chen, Wei Xia, Song Xue, Hang Huang, Qi Lin, Yi Liu, Tongtong Liu, Yiqun Zhang, Panwang Zhang, Jianfei Wang, Yining Yang, Baijun Dong, Zhixian Yu

**Affiliations:** ^1^ Department of Urology, The First Affiliated Hospital of Wenzhou Medical University, Wenzhou City, China; ^2^ Department of Urology, The First Affiliated Hospital of Nanjing Medical University, Nanjing, China; ^3^ Department of Urology, General Hospital of Eastern Theater Command, Nanjing, China; ^4^ Department of Obstetrics and Gynecology, The Second Affiliated Hospital of Wenzhou Medical University, Wenzhou City, China; ^5^ Research Institute, GloriousMed Clinical Laboratory Co., Ltd., Shanghai, China; ^6^ Department of Urology, Renji Hospital, School of Medicine, Shanghai Jiao Tong University, Shanghai, China

**Keywords:** prostate cancer in China, *BRCA1* and *BRCA2* germline mutations, somatic mutation, *BRCA* variants, pathogenic rate

## Abstract

Recent studies have indicated that prostate cancer (PCa) with *BRCA2* mutations is more aggressive. However, these reports mostly focused on Caucasus populations, and large-scale studies on *BRCA* mutations in Chinese PCa populations remain limited. Herein, we screened, from multiple centers in China, a total of 172 patients with PCa carrying *BRCA1/2* germline mutations. The variant distribution and type, associated somatic variant, and frequency of the *BRCA* germline variants in these patients were analyzed retrospectively. We found that Chinese patients with PCa carrying *BRCA1/2* germline mutations were diagnosed at an earlier age, i.e., 67 years (range, 34–89 years), and most had metastatic castration-resistant PCa (mCRPC) (54.65%, 94/172). The top three *BRCA* variants were frameshift, missense, and splicing variants. The overall pathogenic rates of the *BRCA1* and *BRCA2* variants were 17.46% (11/63) and 56.55% (82/145), respectively. Among the somatic mutations associated with *BRCA2* germline mutations, the highest frequency was for *FOXA1* (circulating tumor DNA [ctDNA] sequencing, 7.4%; tissue samples, 52%) and *NCOR2* mutations (ctDNA sequencing, 7.4%; tissue samples, 24%); *TP53* was the dominant somatic mutation associated with *BRCA1* germline mutations (ctDNA sequencing, 25%; tissue samples, 17%). Ultimately, in Chinese patients, PCa with *BRCA1*/2 germline mutations tends to be more aggressive. Compared with *BRCA1*, *BRCA2* has a higher frequency of germline pathogenic mutations. *FOXA1*, *NCOR2*, and *TP53* somatic mutations associated with higher *BRCA1/2* germline pathogenic mutations. Our description of *BRCA* germline mutations in the Chinese PCa patients provides more reference data for the precise diagnosis and treatment of Chinese PCa patients.

## Introduction

Prostate cancer (PCa) is the highest-incidence male genitourinary system malignancy, but there are great differences in the incidence and mortality between patients in China and in other countries (1). Although PCa incidence in China is far lower than that in the western countries, it has been increasing by the year in recent years with lifestyle changes and improved cancer diagnosis levels (2). Genetics are one of the most important factors in PCa, especially in men with a family history of malignancy. Although the clinical significance of common genetic variants associated with PCa risk remains unclear, breast cancer susceptibility gene 1 (*BRCA1*) and *BRCA2* are closely associated with PCa invasiveness and prognosis (3).

*BRCA* is a co-regulator of androgen receptor (AR), and the AR-mediated signaling pathway plays an important role in PCa occurrence and development. Some clinical studies have shown that patients with *BRCA1* and *BRCA2* mutations are more likely to have lymph node involvement or distant metastases when diagnosed, and shorter disease-free survival than patients with wild-type *BRCA* (4). Several large clinical studies have found that patients with metastatic castration-resistant PCa (mCRPC) with somatic or germline variants of the DNA damage repair (DDR) genes (especially *BRCA1*/2) may be sensitive to poly-ADP-ribose polymerase (PARP) inhibitors (PARPi) (5, 6). The PROfound phase III clinical study revealed that patients with PCa with homologous recombination repair (HRR) gene mutations can benefit from olaparib monotherapy; in particular, the risk of radiographic progression (66%) or death could be reduced in patients with *BRCA1*/2 and *ATM* mutations (7).

Therefore, it is necessary to test for *BRCA* mutations in patients with PCa, especially men with a family history of malignancy. Further, the consensus of Chinese experts on genetic testing for patients with PCa recommends testing for *BRCA2* and *BRCA1* germline mutations in patients with high-risk, locally progressive, and metastatic PCa (8). However, research data on Chinese patients with PCa carrying *BRCA1*/2 germline mutations are relatively scarce so far.

Active surveillance of *BRCA* mutation carriers is not safe, even for low-risk patients. When PCa is diagnosed in *BRCA* mutation carriers, radical treatment should be performed as early as possible. Currently, reports related to *BRCA* germline mutations in patients with PCa are mainly concentrated in foreign populations, while studies in Chinese populations are very limited. To reveal the status of *BRCA* germline mutations in the Chinese PCa population, 172 patients with PCa with *BRCA* germline mutations diagnosed at multiple centers were screened for: (1) retrospective statistical analysis of the Chinese PCa population with *BRCA* germline mutations in different pathological stages; (2) exploring the variant distribution and type, and the associated somatic mutations of the *BRCA1*/2 mutations. Ultimately, this study provides more reference data for the precise diagnosis and treatment of patients with PCa in China and can be used to guide clinical decision-making in PCa.

## Methods

### Patients and Samples

We conducted a retrospective study of 172 PCa patients with *BRCA1/2* germline alterations (**Table S1**) and 312 PCa patients without *BRCA* germline mutations (**Table S2**) who underwent genomic profiling with a hybridization capture-based next-generation sequencing (NGS) assay between February 2018 and June 2020 collected from the database of GloriousMed Technology Co., Ltd. (Shanghai, China). These patients were mostly from four hospitals (Renji Hospital of Shanghai Jiaotong University School of Medicine, The First Affiliated Hospital of Wenzhou Medical University, General Hospital of Eastern Theater Command, The First Affiliated Hospital of Nanjing Medical University). The study was approved by the Committee for Ethics of the First Affiliated Hospital of Wenzhou Medical University and informed consent was obtained from each patient. We collected 123 circulating tumor DNA (ctDNA) samples from and 59 biopsied tumor tissue samples from the 172 patients (**Table S1**).

### DNA Sequencing and Bioinformatics

The samples underwent NGS testing at GloriousMed Clinical Laboratory Co., Ltd. Cell-free DNA (cfDNA, from plasma), tumor formalin-fixed, paraffin-embedded (FFPE) DNA, and genomic DNA (gDNA, from white blood cells) were extracted according to standard procedures using a QIAamp Circulating Nucleic Acid Kit (Qiagen), QIAamp DNA FFPE Tissue Kit (Qiagen), and Blood Genomic DNA Mini Kit (cwbiotech), respectively. From each sample, 200–500 ng FFPE DNA, 20–100 ng cfDNA, or 500 ng gDNA were used for library preparation and quantification according to KAPA HyperPrep protocols (KAPA). The genes’ coding regions were captured using custom-designed DNA enrichment panels (50/66/620/642 panels). For analysis, we focused on the common 50 genes (**Supplementary Table 2**). Library pools (5–6) were hybridized to the capture panel according to standard procedures. Then, the libraries were purified and quantified using AMPure XP (Beckman Coulter) and a Qubit™ dsDNA HS Assay Kit (Thermo Fisher Scientific). The final libraries were sequenced on Illumina NextSeq 500 (75-bp paired-end reads [PE75]) or NovaSeq 6000 (PE150) instruments.

### Quality Control and Variant Calling

The raw data were trimmed using Trimmomatic (9). Then, the reads were aligned with the human reference genome (hg19) using Burrows-Wheeler Aligner (10). Duplicated reads were removed using Picard (http://broadinstitute.github.io/picard/). Mapped reads were realigned to the genome using Genome Analysis Tool Kit (GATK) (11). Germline mutations were called using GATK’s HaplotypeCaller (11) with a paired workflow. Variants were then annotated using ANNOVAR (12) and an in-house-developed code. The human identity concordance of the paired samples was verified using an in-house script. Germline mutations considered deleterious (frameshift insertions, nonsense/stop-gains, splice site variants, deletions, or reported as pathogenic or likely pathogenic in the ClinVar database) were included for analysis. Here, “pathogenic alterations” includes pathogenic or likely pathogenic alterations; “non-pathogenic” represents variants of uncertain significance (VUS).

### Statistical Analysis

The assessment of clinical characteristics between different cohorts, including age at diagnosis, Gleason score, et al., were based on the Wilcoxon rank sum test. Graphpad Prism V8 (GraphPad Software, Inc.) and R v3.6.1 (www.R-project.org) were used for data analysis. A two-sided P value <0.05 was considered significant.

## Results

### Analysis of the Patients’ Characteristics

The 172 patients with *BRCA* germline mutations comprised patients diagnosed with PCa (NA), localized prostate cancer (LAPC), metastatic hormone-sensitive prostate cancer (mHSPC), or mCRPC (**Table 1**). Castration resistance was defined according to the European Association of Urology (EAU) Guidelines on Prostate Cancer (2021 edition). Significant difference was found in median age between those with and without *BRCA1* mutation (69 years; range, 53–89 years *vs*. 66 years; range, 44–98 years, p < 0.05), but not in *BRCA2* mutation(65.5 years; range, 34–85 years *vs*. 66 years; range, 44–98 years, p > 0.05). Overall, there was no significant difference in PSA value (13.6; range, 0–1000 *vs*. 0; range, 0–5000, p > 0.05) and Gleason score (p > 0.05) between the patients with and without *BRCA* mutation. The baseline comparison between the patients with pathogenic and non-pathogenic BRCA1/2 mutations was performed, significant difference was found in median age between them (65 years; range, 34-82 years *vs*. 67.5 years; range, 53-89 years, p < 0.05). Similarly, there was no significant difference in PSA value (6.95; range, 0–1000 *vs*. 10.85; range, 0–905, p > 0.05) and Gleason score (p > 0.05) between the patients with pathogenic and non-pathogenic BRCA1/2 mutations.

**Table 1:** Summary of clinical characteristics and a comparison between patients with *BRCA1/2* mutation and without *BRCA1/2* mutation (wild).

Baseline inf.		Wild vs. (n=312)	BRCA1+ (n=56)	P value	BRCA2+ (n=119)	P value	BRCA1/2+ (n=172)	P value	BRCA1/2+		
									Pathogenic (n=79)	Non-pathogenic (n=93)	P value
Median age (yr)		66 (44-98)	69(53-89)	0.046	65.5 (34-85)	0.244	67(34-89)	0.096	65 (34-82)	67.5 (53-89)	0.002
Stage	NA^a^	35 (11%)	7 (12.5%)		18 (15.1%)		26 (15.1%)		11 (13.9%)	8 (8.6%)	
	LAPC^b^	35 (11%)	10 (17.9%)		21 (17.6%)		30 (17.4%)		9 (11.4%)	22 (23.7%)	
	mHSPC^c^	88 (28%)	11 (19.6%)		17 (14.3%)		27 (15.7%)		12 (5.2%)	16 (17.2%)	
	mCRPC^d^	154 (49%)	29 (51.8%)		65 (54.6%)		90 (52.3%)		47 (59.5%)	47 (50.5%)	
PSA	Median	0 (0-5000)	15.7 (0-905)	0.801	11.5 (0-1000)	0.696	13.6 (0-1000)	0.623	6.95 (0-1000)	10.85 (0-905)	0.195
	0-10	195 (63%)	21 (37.5%)		46 (38.7%)		65 (37.8%)		34 (43.0%)	35 (37.6%)	
	11-20	9 (3%)	7 (12.5%)		7 (5.9%)		13 (7.6%)		3 (3.8%)	10 (10.8%)	
	21-100	52 (17%)	16 (28.6%)		19 (16%)		32 (18.6%)		15 (19.0%)	29 (31.2%)	
	>100	56 (18%)	7 (12.5%)		24 (20.2%)		34 (19.8%)		13 (16.5%)	11 (11.8%)	
	NA		5 (8.9%)		22 (18.5%)		28 (16.3%)		14 (17.7%)	8 (8.6%)	
Gleason Score	6	4 (1%)		0.555		0.809		0.362			0.063
	7	38 (12%)	8 (14.3%)		15 (12.6%)		23 (13.4%)		8 (10.1%)	15 (16.1%)	
	8	91 (29%)	10 (17.9%)		17 (14.3%)		27 (15.7%)		7 (8.9%)	20 (21.5%)	
	9	136 (44%)	17 (30.4%)		30 (25.2%)		47 (27.3%)		26 (32.9%)	25 (26.9%)	
	10	29 (9%)			3 (2.5%)		3 (1.7%)		2 (2.5%)	1 (1.1%)	
	NA	14 (4%)	21 (37.5%)		53 (44.5%)		72 (43.0%)		36 (45.6%)	32 (34.4%)	

a NA, not available.

b LAPC, Localized prostate cancer.

c mHSPC, metastatic hormone-sensitive prostate cancer.

d mCRPC, metastatic castration resists prostate cancer.

### Frequency and Comparative Analysis of Patients With *BRCA* Germline Mutations

**Figure 1:**
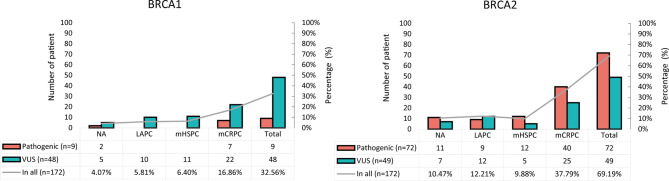
Frequency distribution of pathogenic and non-pathogenic *BRCA1* and *BRCA2* mutations in patients with PCa. VUS, variants of uncertain significance.

The frequency distribution of *BRCA1*/2 germline pathogenic and non-pathogenic (VUS) mutations differed significantly (**Figure 1**). *BRCA1* germline mutations were mainly VUS (27.91%, 48/172) while *BRCA2* germline mutations were mainly pathogenic (41.86%, 72/172). The frequency of *BRCA2* germline mutations in our cohort (69.19%, 121/172) was much higher than that of *BRCA1* germline mutations (32.56%, 57/172) (**Table 1**). Based on pathological stage, *BRCA1* and *BRCA2* mutations were mainly found in mCRPC, especially *BRCA2*, with a frequency of 16.86% (29/172) and 37.79% (65/172), respectively. While it was similar between LAPC and mHSPC, *BRCA1* was 5.81% (10/172) and 6.4% (11/172), *BRCA2* was 12.21% (21/172) and 9.88% (17/172).

### Genetic Distribution of *BRCA1/2* Variants

**Figure 2:**
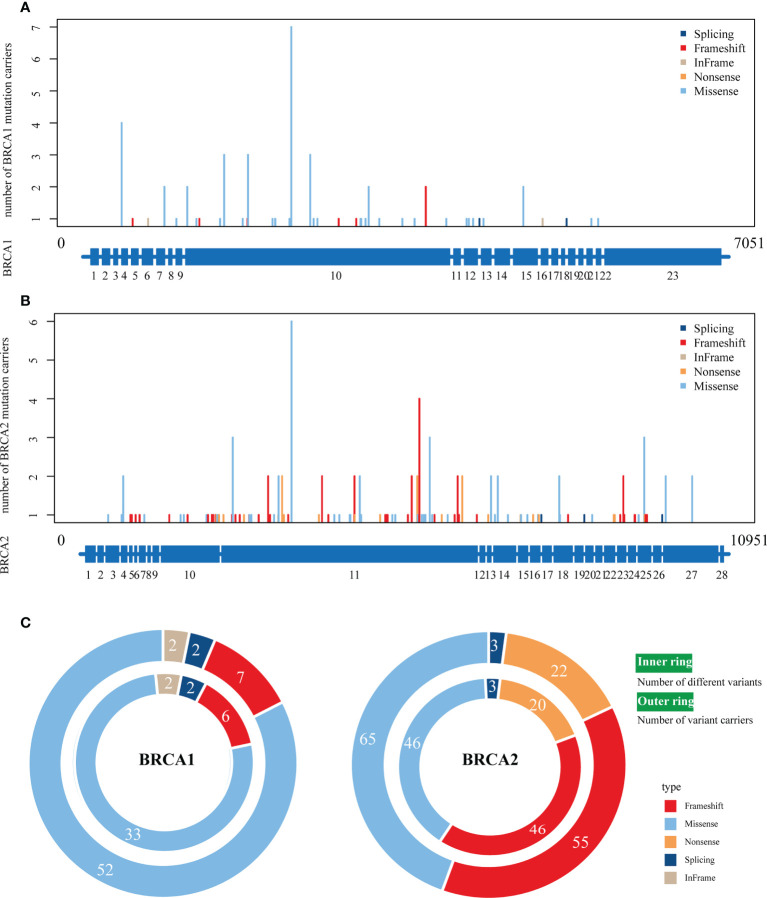
The distribution of various BRCA1/2 variant types on full‐length BRCA1/2 exons. **(A, B)**, The distribution of four variant types on *BRCA1* **(A)** and *BRCA2* **(B)**. The scheme of exons (blue bar at the bottom of each panel) is shown as the reference. **(C)** The number of different variants (inner ring) and number of variant carriers (outer ring). Colors represent different variant types.

The overall analysis showed that the *BRCA1*/2 variants were distributed in most exon regions of the *BRCA1* and *BRCA2* genes, and no new hot spot variants were found (**Figure 2** and **Figure S1**). A total of 208 *BRCA* germline variants were identified: 63 and 145 in *BRCA1* and *BRCA2*, respectively. c.2726A>T (p.N9091) was the most frequently mutated variant (3.37%, 7/208). Most of the variants occurred only once (58.65%, 122/208); 41.35% (86/208) of the variants with >1 occurrence were mainly distributed in mCRPC (55/86). Among the 63 *BRCA1* variants, c.2726.4>T (p.N9091) was the most common (7/63), while c.5722-5723DELCT (p.L1908FS) was the most common (5/145) among the 145 *BRCA2* variants.

In the variant type analysis, frameshift, missense, and splicing were the common *BRCA1* and *BRCA2* variants. Frameshift and missense were the most advantageous variants, occurring in 90.7% (132/145) and 80% (50/63) of *BRCA1* and *BRCA2* variants, respectively. The difference was that in-frame and nonsense variants only appeared in *BRCA2* and *BRCA1*, respectively.

### Statistical Analysis of *BRCA* Variants

**Figure 3:**
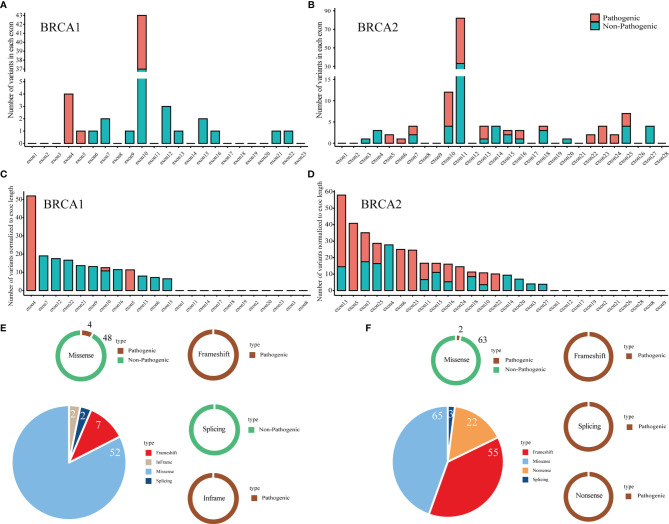
Interpretation of pathogenicity and distribution of pathogenic and non-pathogenic variants in full-length BRCA1/2 genes. **(A, B)** The number of variants in each exon of the BRCA1 **(A)** and BRCA2 **(B)** gene. **(C, D)**, The number of variants normalized to exon length for BRCA1 **(C)** and BRCA2 **(D)**. **(E, F)** The relative ratio of each type of pathogenicity in each type of variant in BRCA1 **(E)** and BRCA2 **(F)**.

Here, we report the distribution of the pathogenic and non-pathogenic variants in the major exons of *BRCA1* and *BRCA2* (**Figures 3A, B**). There were 54.55% (6/11) and 69.51% (57/82) pathogenic variants in *BRCA1* exon 10 and *BRCA2* exon 10/11, respectively. The number of variants per exon was normalized according to the exon length (**Figures 3C, D**). Exon 4 and exon 13 had the most variants in *BRCA1* and *BRCA2*, respectively. Exon 4 and exon 5 had the most pathogenic variants in *BRCA1* and *BRCA2*, respectively. Among all variants, the overall pathogenic rates for *BRCA1* and *BRCA2* were 17.46% (11/63) and 56.55% (82/145), respectively (**Figures 3E, F**). The frameshift variants were pathogenic both in *BRCA1* and *BRCA2*. Similarly, missense variants also showed the same trend in *BRCA1* and *BRCA2*. The difference was that splicing variants were non-pathogenic and pathogenic in *BRCA1* and *BRCA2*, respectively.

### Somatic Mutation Analysis of Patients With *BRCA* Germline Pathogenic Mutations

**Figure 4:**
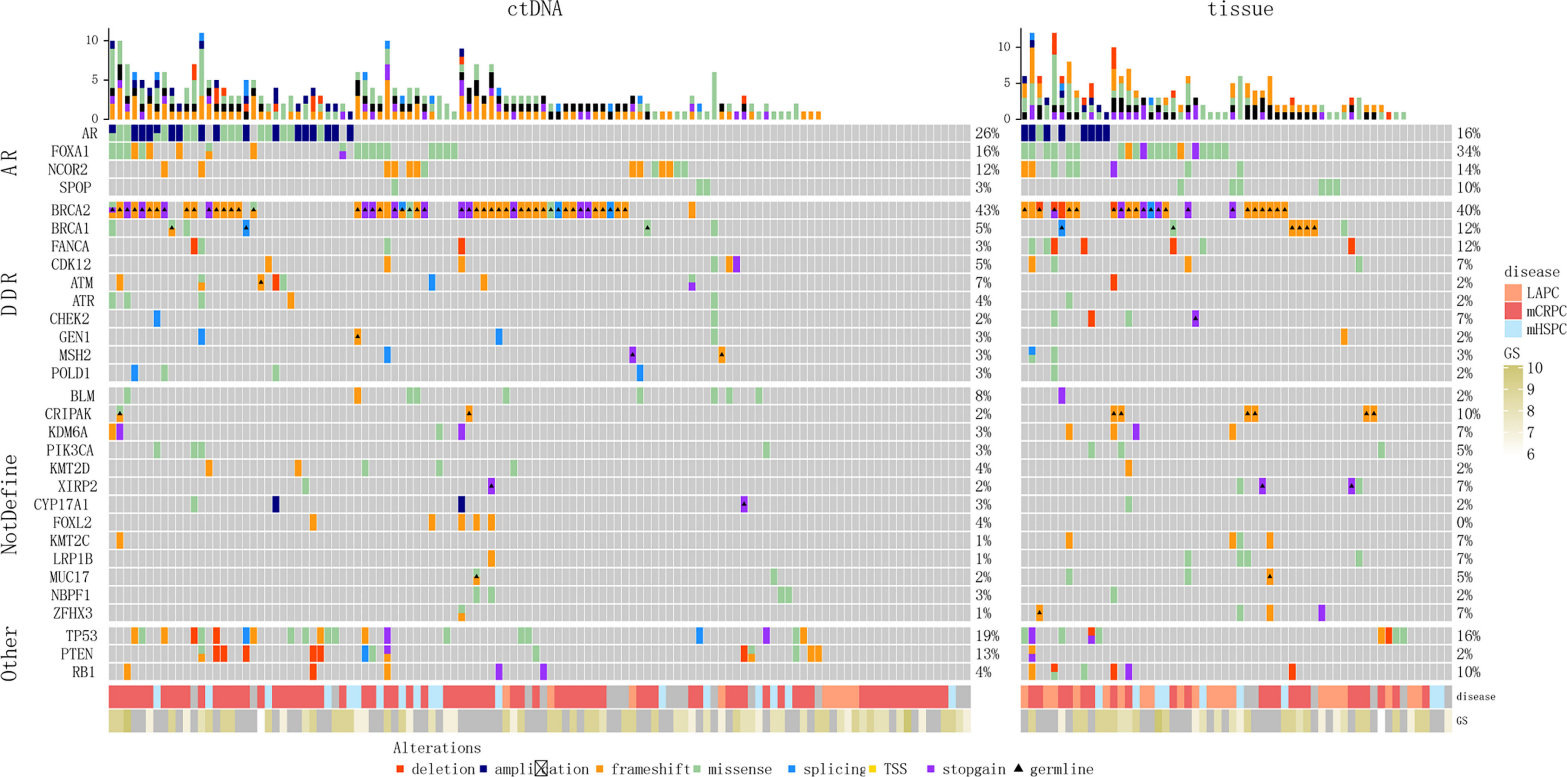
Genomic landscape of patients with PCa with *BRCA* germline pathogenic mutations. **(A)** Blood samples. **(B)** Tissue samples. TSS, translation start site.

We identified somatic alterations in the AR pathway genes, DDR pathway genes, and tumor suppressor genes (*TP53*/RB1) in the patients (**Figure 4**). *AR* (26%, 32/123), *TP53* (20%, 25/123), *FOXA1* (15%, 18/123), *NCOR2* (12%, 15/123), and *PTEN* (10%, 12/123) were the top five somatic mutation genes associated with ctDNA sequencing. In the tissue samples, the top seven somatic mutation genes were *FOXA1* (34%, 20/59), *TP53* (15%, 9/59), *AR* (15%, 9/59), *NCOR2* (14%, 8/59), *FANCA* (12%, 7/59), *RB1* (12%, 7/59), and *SPOP* (10%, 6/59). Among the somatic mutations associated with *BRCA2* germline mutations, the most frequent were *FOXA1* (ctDNA sequencing, 7.4% [4/54]; tissue samples, 52% [11/21) and *NCOR2* (ctDNA sequencing, 7.4% [4/54]; tissue samples, 24% [5/21]) mutations; *TP53* was the dominant somatic mutation associated with *BRCA1* germline mutations (ctDNA sequencing, 25% [1/4]; tissue samples, 17% [1/6]).

Further, there were some differences in the blood and tissue profiles, so we conducted consistency analysis on mutation data from 10 patients using both tissue and matched blood samples (**Figure S2** and **Table S3**). Most of the 10 patients had a relatively high degree of consistency between the mutations in the tissue and matched blood samples (e.g. Patients #2, 6, 9). However, the sample size (10 patients) was small, which limited consistent comparison of the occurrence frequency of the related genes in specific tissue and blood pathways.

## Discussion

### Differences in BRCA Germline Mutations in Different Populations

Research targeting *BRCA1*/2 mutations has received increasing attention in recent years, in part because of the success of PARPi in clinical studies. While these studies were mostly focused on foreign populations, there have been few studies on Chinese populations. Studying *BRCA1*/2 mutations in the Chinese PCa population will enable more comprehensive understanding of *BRCA1*/2 mutations in this population, and further insightful analysis of the characteristics of these mutations will ultimately provide a more optimal treatment plan for patients.

Although our cohort was smaller than that in another prospective study of *BRCA1* and *BRCA2* germline pathogenic mutations in a Chinese population (172 *vs.* 316) (13), our study has a larger Chinese cohort with *BRCA1* and *BRCA2* germline pathogenic mutations (9 *vs*. 2, 72 *vs*. 20). The *BRCA2* germline mutation carriers in the present study were at an earlier age, i.e., 67 years (range, 34–89 years), which was similar to previous reports of patients with PCa with *BRCA2* mutations having an earlier age of diagnosis (14). Furthermore, most of the clinical stages were concentrated in the mCRPC stage and had high Gleason scores, and the frequency of *BRCA1* and *BRCA2* germline mutations during the metastatic PCa (MPC) stage (mHSPC and mCRPC) was higher than that in the localized stage. These results confirm that *BRCA1*/2 mutation carriers are more likely to have lymph node involvement and distant metastases (15). *BRCA*-positive PCa populations often have higher Gleason scores (≥8) and higher tumor-node-metastasis (TNM) stage (15). These findings could provide more comprehensive evidence for novel endocrine therapy treatments for PCa. New endocrine therapy has a better effect on *BRCA* mutation carriers compared with non-carriers in the mCRPC population, and PCa populations with *BRCA1* or *BRCA2* mutations could benefit from abiraterone or enzalutamide treatment (16).

### Analysis of BRCA Variants

Understanding the distribution of pathogenic variants in *BRCA1*/2 in key domains (exons or introns) and the role of each specific variant is of great significance for PCa treatment. Here, *BRCA1*/2 variant analysis revealed no distinct hotspot mutation. In patients with PCa, the *BRCA2* gene has a higher risk of mutations in the c.756-c.1000 and c.7914+ regions (17, 18). However, our results yield no similar conclusions: the frequency of *BRCA2* mutation was 16.55% (24/145) in the c.7914+ region, and was 8.97% (13/145) in the in c.756-c.1000 region.

Notably, each variant type showed different characteristics. Here, the top three *BRCA* variants were frameshift, missense, and splicing variants. Meta-analyses investigating the presence of *BRCA* genes in patients with cancer found that the missense variant was the most frequent in patients with *BRCA1* and *BRCA2* variants (19). However, our results show that the frameshift variant was the most frequent in *BRCA2* variant carriers, and that all of the variants were pathogenic. Therefore, it is necessary to develop new specific tests for exon or intron–exon boundaries for more accurate PCa clinical diagnosis and treatment.

### Differences Between BRCA1 and BRCA2 Mutations

We collected *BRCA1*/2 gene germline mutation data from the Chinese PCa population, and found that the *BRCA2* mutation frequency (69.19%) was much higher than that of *BRCA1* (32.56%), and that most mutations occurred at the MPC (mHSPC and mCRPC) stage. This suggests that there might be different tumor gene expression patterns in *BRCA2*. This is similar to previous studies reporting that the characteristics of *BRCA2* mutated tumors were more similar to those of mCRPC than of LAPC (20–22). In 6,902 men with *BRCA1* or *BRCA2* mutations who developed cancer, especially breast, prostate, and pancreatic cancer, and multiple primary tumors, there was an association with a higher rate of *BRCA2* mutations (23). Moreover, clinical trial data (TRITON2 and PROfound) have shown that patients with *BRCA2* mutations could benefit more from PARPi than those with *BRCA1* mutations (24). These results suggest that patients with PCa with *BRCA2* mutations might receive higher prognostic benefit than *BRCA1* carriers.

There is an association between patients with PCa with *BRCA1*/2 mutations carrying other mutations (e.g., *TP53*) with poorer prognosis and PARPi sensitivity (25). The TRITON2 study observed 62% and 42% *BRCA1* and *BRCA2* mutation carriers with *TP53* mutations, respectively (26). The cBioPortal database, which contains publicly available genomic information, shows that harmful *TP53* mutations are more common in patients with PCa carrying *BRCA1* mutations than in patients carrying *BRCA2* mutations (39% *vs*. 23%) (26). In the present study, ctDNA sequencing showed that the *TP53* mutation frequency in the somatic mutant along with *BRCA1* germline mutations was much higher than that of *BRCA2* germline mutation (25% [1/4] and 5.6% [3/54]). In tissue sequencing, the frequency was 17% (1/6) and 5% (1/21), respectively.

We also found that, except *TP53*, *FOXA1* and *NCOR2*, along with *BRCA1* germline mutations, were more frequent than *BRCA2* germline mutations. Tumors with *FOXA1* mutations accompanied by higher Gleason scores, shorter biochemical relapse time, and faster metastatic disease progression (27). *NCOR2* could interact with AR, thereby inhibiting the transcriptional activity of AR (28, 29). Recent studies have also found that patients with *FOXA1* and *NCOR2* mutations had poor prognosis (22). This evidence suggests that *FOXA1* and *NCOR2* somatic mutations may affect disease progression in patients with *BRCA* germline mutation. However, our results lack follow-up information for the patients, and the relationship between *FOXA1* and *NCOR2* mutations and the prognosis of patients carrying *BRCA* germline mutations should be explored in the future.

Our study has some limitations. Our data contain many variants of VUS (53.85%, 112/208), which prevents elucidation of the pathogenicity of some mutations, consequently delaying the selection of appropriate therapies. Therefore, new database updates or more information mining of mutations are needed. In addition, the panel we used can only capture exon regions and may have missed some meaningful intron mutations.

## Conclusions

Our results reveal that PCa with *BRCA2* germline mutations is highly aggressive in Chinese patients. The frequency of *BRCA1* and *BRCA2* germline mutations was significantly different, *FOXA1*, *NCOR2*, and *TP53* somatic mutations associated with higher *BRCA1/2* germline pathogenic mutations. Our results suggest that early genetic testing should be actively recommended for patients with PCa family inheritance, which could provide more accurate data support for them to obtain better treatment.

## Data Availability Statement

The data analyzed in this study is subject to the following licenses/restrictions: The data in the article relates to Chinese human genetic data, so it is not disclosed. Requests to access these datasets should be directed to gylchen0@qq.com.

## Ethics Statement

The studies involving human participants were reviewed and approved by The First Affiliated hospital of Wenzhou Medical University. The patients/participants provided their written informed consent to participate in this study.

## Author Contributions

Conception and design: WC, WX, SX, BD, and ZY. Acquisition of data (acquired and managed patients, provided facilities, etc.): WX, SX, HH, QL, YL, PZ, BD, and ZY. Analysis and interpretation of data (e.g., statistical analysis, biostatistics, and computational analysis): TL, YZ, JW, and YY. Writing, review, and/or revision of the manuscript: TL, JW, and YY. Administrative, technical, or material support (i.e., reporting or organizing data, constructing databases): WX, SX, HH, QL, and BD. Study supervision: BD and ZY. All authors contributed to the article and approved the submitted version.

## Funding

This research was supported by Zhejiang Provincial Natural Science Foundation of China (No. LY20H160013), Zhejiang Provincial Traditional Chinese Medicine Scientific Research Fund (No. 2022ZB215), Zhejiang Province Medical and health science and Technology project (No. 2022KY199), Zhejiang Province Medical and health science and Technology project (No. 2020KY185), and Clinical Research Special Fund of Wu Jieping Medical Foundation (320.6750.2020-14-6).

## Conflict of Interest

Authors TL, YZ, PZ, JW and YY were employed by company GloriousMed Clinical Laboratory Co., Ltd.

The remaining authors declare that the research was conducted in the absence of any commercial or financial relationships that could be construed as a potential conflict of interest.

## Publisher’s Note

All claims expressed in this article are solely those of the authors and do not necessarily represent those of their affiliated organizations, or those of the publisher, the editors and the reviewers. Any product that may be evaluated in this article, or claim that may be made by its manufacturer, is not guaranteed or endorsed by the publisher.

## Supplementary Material

Supplementary Figure 1 Alterations in the *BRCA* gene. **(A)** *BRCA1* mutations were primarily missense and truncating. **(B)** *BRCA2* mutations were primarily truncating, in-frame, and missense.

Supplementary Figure 2 Concordance of mutation calls between ctDNA samples and paired tumor tissues in the 10 patients.

## References

[1] Khazaei Z Jarrahi AM Momenabadi V Ghor F Goodarzi E. Global Cancer Statistics 2018: Globocan Estimates Of Incidence And Mortality Worldwide Stomach Cancers And Their Relationship With The Human Development Index (HDI). Adv Hum Biol (2019) 9(3):e1257. doi: 10.4103/2321-8568.262891

[2] Chen W Zheng R Baade PD Zhang S Zeng H Bray F. Cancer Statistics in China, 2015. CA Cancer J Clin (2016) 66(2):115–32. doi: 10.3322/caac.21338 26808342

[3] Ferlay J Soerjomataram I Dikshit R Eser S Mathers C Rebelo M. Cancer Incidence and Mortality Worldwide: Sources, Methods and Major Patterns in GLOBOCAN 2012. Int J Cancer (2015) 136(5):E359–86. doi: 10.1002/ijc.29210 25220842

[4] Gallagher DJ Cronin AM Milowsky MI Morris MJ Bhatia J Scardino PT. Germline BRCA Mutation Does Not Prevent Response to Taxane-Based Therapy for the Treatment of Castration-Resistant Prostate Cancer. BJU Int (2012) 109(5):713–9. doi: 10.1111/j.1464-410X.2011.10292.x PMC3971723 21756279

[5] Pomerantz MM Spisak S Jia L Cronin AM Csabai I Ledet E. The Association Between Germline BRCA2 Variants and Sensitivity to Platinum-Based Chemotherapy Among Men With Metastatic Prostate Cancer. Cancer (2017) 123(18):3532–9. doi: 10.1002/cncr.30808 PMC5802871 28608931

[6] Kaufman B Shapira-Frommer R Schmutzler RK Audeh MW Friedlander M Balmana J. Olaparib Monotherapy in Patients With Advanced Cancer and a Germline BRCA1/2 Mutation. J Clin Oncol (2015) 33(3):244–50. doi: 10.1200/JCO.2014.56.2728 PMC6057749 25366685

[7] Di Lorenzo G Autorino R. Re: Olaparib for Metastatic Castration-Resistant Prostate Cancer. Eur Urol (2020) 78(5):767–8. doi: 10.1016/j.eururo.2020.06.011 32624284

[8] Zhu Y. Chinese Expert Consensus on Genomic Testing of Prostate Cancer Patients (the 2020 Edition). China Oncol (2020) 30(07):551–60. doi: 10.19401/j.cnki.1007-3639.2020.07.011

[9] Bolger AM Lohse M Usadel B. Trimmomatic: A Flexible Trimmer for Illumina Sequence Data. Bioinformatics (2014) 30(15):2114–20. doi: 10.1093/bioinformatics/btu170 PMC4103590 24695404

[10] Li H Durbin R. Fast and Accurate Short Read Alignment With Burrows-Wheeler Transform. Bioinformatics (2009) 25(14):1754–60. doi: 10.1093/bioinformatics/btp324 PMC2705234 19451168

[11] McKenna A Hanna M Banks E Sivachenko A Cibulskis K Kernytsky A. The Genome Analysis Toolkit: A MapReduce Framework for Analyzing Next-Generation DNA Sequencing Data. Genome Res (2010) 20(9):1297–303. doi: 10.1101/gr.107524.110 PMC2928508 20644199

[12] Wang K Li M Hakonarson H. ANNOVAR: Functional Annotation of Genetic Variants From High-Throughput Sequencing Data. Nucleic Acids Res (2010) 38(16):e164–4. doi: 10.1093/nar/gkq603 PMC2938201 20601685

[13] Bratt O Loman N. Clinical Management of Prostate Cancer in Men With BRCA Mutations. Eur Urol (2015) 68(2):194–5. doi: 10.1016/j.eururo.2014.11.005 25465969

[14] Page EC Bancroft EK Brook MN Assel M Hassan Al Battat M Thomas S. Interim Results From the IMPACT Study: Evidence for Prostate-Specific Antigen Screening in BRCA2 Mutation Carriers. Eur Urol (2019) 76(6):831–42. doi: 10.1016/j.eururo.2019.08.019 PMC6880781 31537406

[15] Castro E Goh C Olmos D Saunders E Leongamornlert D Tymrakiewicz M. Germline BRCA Mutations Are Associated With Higher Risk of Nodal Involvement, Distant Metastasis, and Poor Survival Outcomes in Prostate Cancer. J Clin Oncol (2013) 31(14):1748–57. doi: 10.1200/JCO.2012.43.1882 PMC3641696 23569316

[16] Antonarakis ES Lu C Luber B Liang C Wang H Chen Y. Germline DNA-Repair Gene Mutations and Outcomes in Men With Metastatic Castration-Resistant Prostate Cancer Receiving First-Line Abiraterone and Enzalutamide. Eur Urol (2018) 74(2):218–25. doi: 10.1016/j.eururo.2018.01.035 PMC6045965 29439820

[17] Nyberg T Frost D Barrowdale D Evans DG Bancroft E Adlard J. Prostate Cancer Risk by BRCA2 Genomic Regions. Eur Urol (2020) 78(4):494–7. doi: 10.1016/j.eururo.2020.05.005 PMC7532700 32532514

[18] Patel VL Busch EL Friebel TM Cronin A Leslie G McGuffog L. Association of Genomic Domains in BRCA1 and BRCA2 With Prostate Cancer Risk and Aggressiveness. Cancer Res (2020) 80(3):624–38. doi: 10.1158/0008-5472.CAN-19-1840 PMC7553241 31723001

[19] Sun P Li Y Chao X Li J Luo R Li M. Clinical Characteristics and Prognostic Implications of BRCA-Associated Tumors in Males: A Pan-Tumor Survey. BMC Cancer (2020) 20(1):994. doi: 10.1186/s12885-020-07481-1 33054725 PMC7556962

[20] Robinson D. Integrative Clinical Genomics of Advanced Prostate Cancer. Cell (2015) 161(5):1215–28. doi: 10.1016/j.cell.2015.05.001 PMC4484602 26000489

[21] Taylor RA Fraser M Livingstone J Espiritu SM Thorne H Huang V. Germline BRCA2 Mutations Drive Prostate Cancers With Distinct Evolutionary Trajectories. Nat Commun (2017) 8:13671. doi: 10.1038/ncomms13671 28067867 PMC5227331

[22] Armenia J Wankowicz SAM Liu D Gao J Kundra R Reznik E. The Long Tail of Oncogenic Drivers in Prostate Cancer. Nat Genet (2018) 50(5):645–51. doi: 10.1038/s41588-018-0078-z PMC6107367 29610475

[23] Silvestri V Leslie G Barnes DR CG Agnarsson BA Aittomaki K. Characterization of the Cancer Spectrum in Men With Germline BRCA1 and BRCA2 Pathogenic Variants: Results From the Consortium of Investigators of Modifiers of BRCA1/2 (CIMBA). JAMA Oncol (2020) 6(8):1218–30. doi: 10.1001/jamaoncol.2020.2134 PMC7333177 32614418

[24] Markowski MC Antonarakis ES. BRCA1 Versus BRCA2 and PARP Inhibitor Sensitivity in Prostate Cancer: More Different Than Alike? J Clin Oncol (2020) 38(32):3735–9. doi: 10.1200/JCO.20.02246 10.1200/JCO.20.02246 PMC7655018 32870734

[25] Abida W Cyrta J Heller G Prandi D Armenia J Coleman I. Genomic Correlates of Clinical Outcome in Advanced Prostate Cancer. Proc Natl Acad Sci USA (2019) 116(23):11428–36. doi: 10.1073/pnas.1902651116 PMC6561293 31061129

[26] Abida W Patnaik A Campbell D Shapiro J Bryce AH McDermott R. Rucaparib in Men With Metastatic Castration-Resistant Prostate Cancer Harboring a BRCA1 or BRCA2 Gene Alteration. J Clin Oncol (2020) 38(32):3763–72. doi: 10.1200/JCO.20.01035 PMC7655021 32795228

[27] Adams EJ Karthaus WR Hoover E Liu D Gruet A Zhang Z. FOXA1 Mutations Alter Pioneering Activity, Differentiation and Prostate Cancer Phenotypes. Nature (2019) 571(7765):408–12. doi: 10.1038/s41586-019-1318-9 PMC6661172 31243370

[28] Perissi V Jepsen K Glass CK Rosenfeld MG. Deconstructing Repression: Evolving Models of Co-Repressor Action. Nat Rev Genet (2010) 11(2):109–23. doi: 10.1038/nrg2736 20084085

[29] Laschak M Bechtel M Spindler KD Hessenauer A. Inability of NCoR/SMRT to Repress Androgen Receptor Transcriptional Activity in Prostate Cancer Cell Lines. Int J Mol Med (2011) 28(4):645–51. doi: 10.3892/ijmm.2011.735 21720703

