# Supplementary figures and images for: Analysis of BRCA Germline Mutations in Chinese Prostate Cancer Patients
Source: Front Oncol. 2022 Feb 17;12:746102. doi: 10.3389/fonc.2022.746102 (PMC8892236; doi:10.3389/fonc.2022.746102)

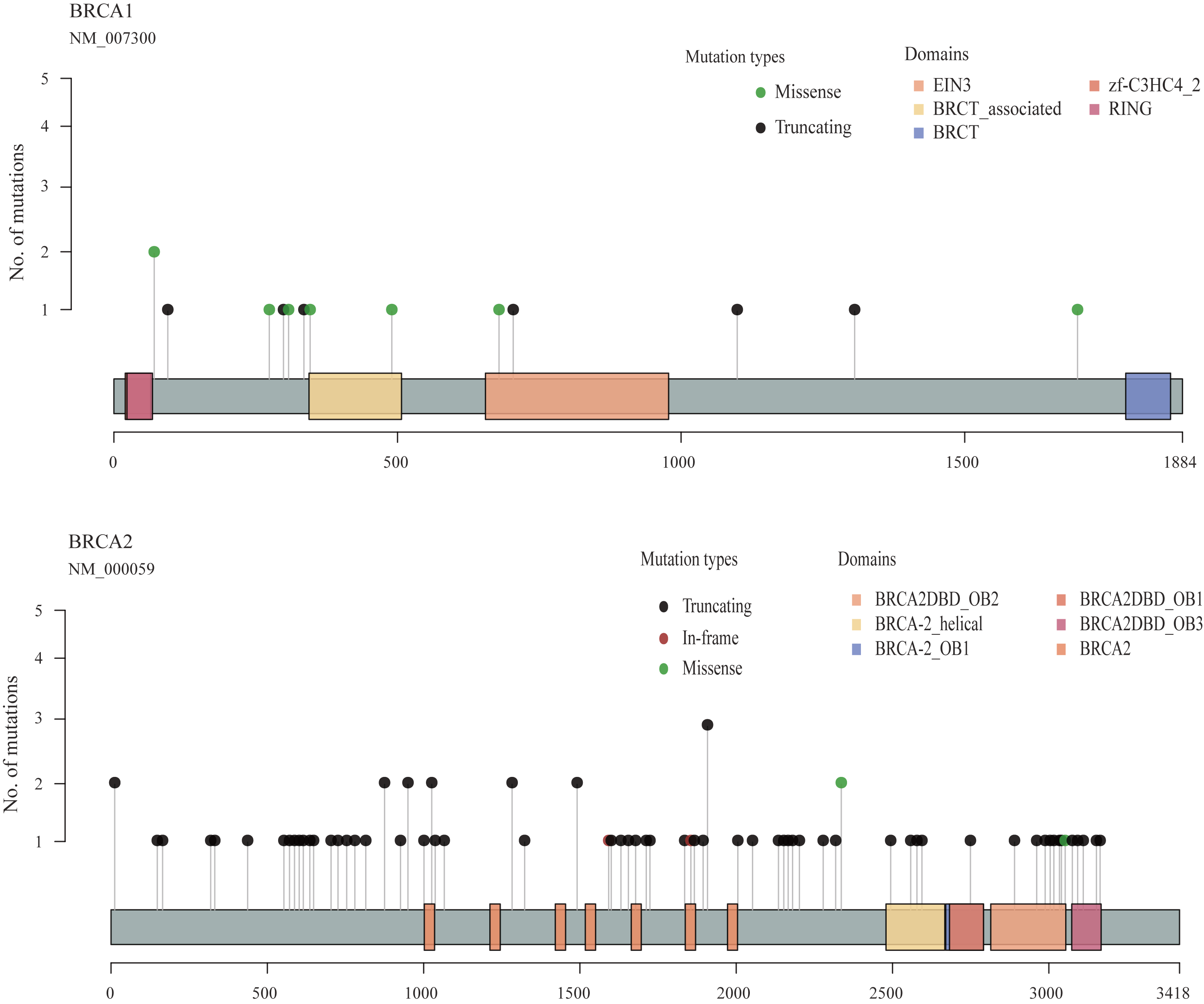

Supplement: Supplementary Figure 1 — Alterations in the BRCA gene. (A) BRCA1 mutations were primarily missense and truncating. (B) BRCA2 mutations were primarily truncating, in-frame, and missense. [file Image_1.tif]

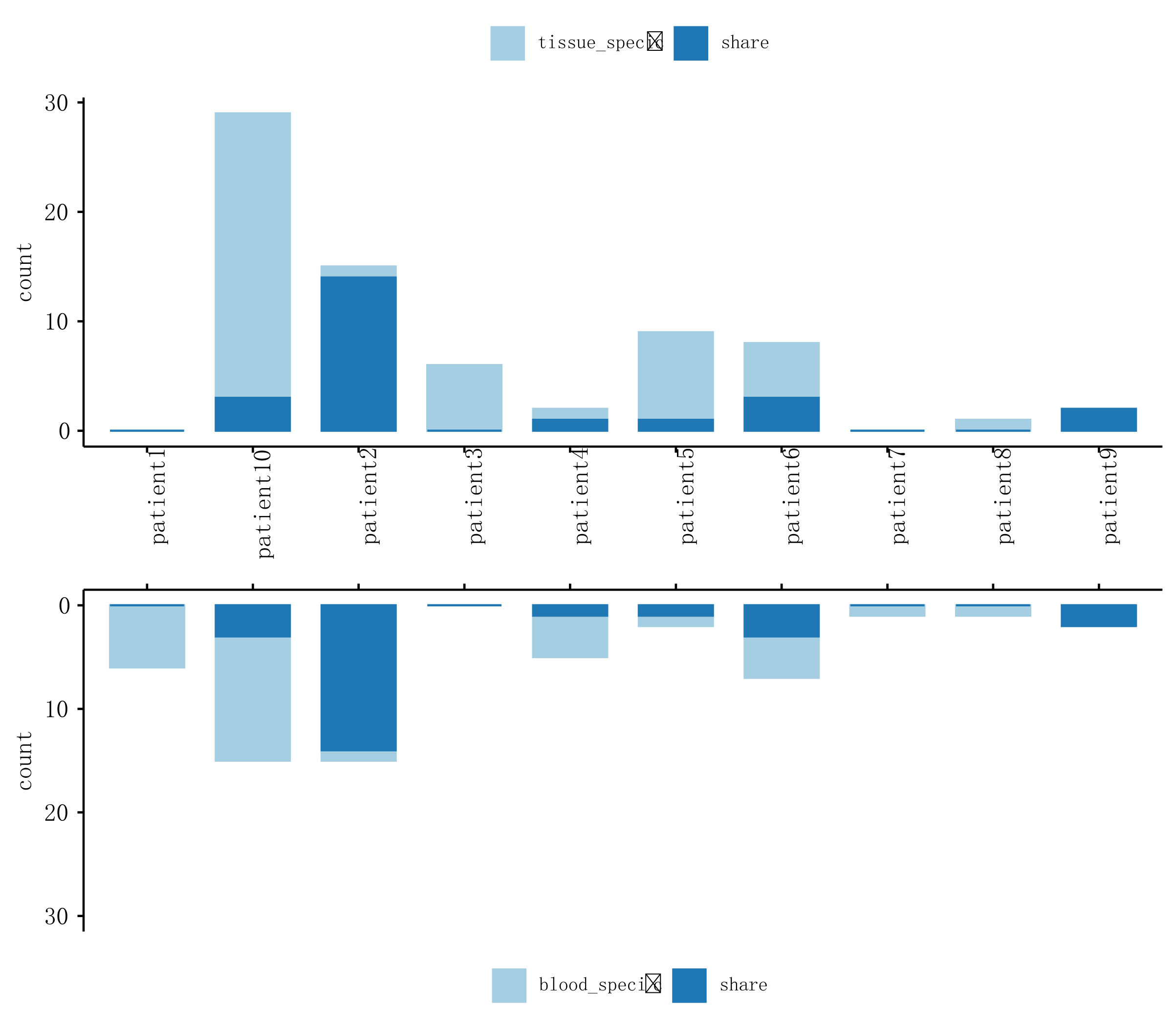

Supplement: Supplementary Figure 2 — Concordance of mutation calls between ctDNA samples and paired tumor tissues in the 10 patients. [file Image_2.tif]
